# Barcoding the largest animals on Earth: ongoing challenges and molecular solutions in the taxonomic identification of ancient cetaceans

**DOI:** 10.1098/rstb.2015.0332

**Published:** 2016-09-05

**Authors:** Camilla Speller, Youri van den Hurk, Anne Charpentier, Ana Rodrigues, Armelle Gardeisen, Barbara Wilkens, Krista McGrath, Keri Rowsell, Luke Spindler, Matthew Collins, Michael Hofreiter

**Affiliations:** 1 BioArCh, Department of Archaeology, University of York, Environment Building, York, North Yorkshire YO10 5DD, UK; 2 Institute of Archaeology, University College London, 31–34 Gordon Square, London WC1H 0PY, UK; 3 CEFE UMR 5175, CNRS, Université de Montpellier, Université Paul-Valéry Montpellier, EPHE – CNRS, Montpellier Cedex 5, France; 4 Archéologie des Sociétés Méditerranéennes, UMR 5140, CNRS, Labex Archimede IA-ANR-11-LABX-0032-01, Université Paul-Valéry Montpellier, 34970 Lattes, France; 5 Dipartimento di Scienze della Natura e del Territorio, Università degli Studi, Sassari, Italy; 6 Institute of Biochemistry and Biology, Faculty of Mathematics and Natural Sciences, University of Potsdam, 14476 Potsdam, Germany

**Keywords:** ancient DNA, archaeozoology, cetaceans, collagen peptide mass fingerprinting, species identification, zooarchaeology by mass spectrometry

## Abstract

Over the last few centuries, many cetacean species have witnessed dramatic global declines due to industrial overharvesting and other anthropogenic influences, and thus are key targets for conservation. Whale bones recovered from archaeological and palaeontological contexts can provide essential baseline information on the past geographical distribution and abundance of species required for developing informed conservation policies. Here we review the challenges with identifying whale bones through traditional anatomical methods, as well as the opportunities provided by new molecular analyses. Through a case study focused on the North Sea, we demonstrate how the utility of this (pre)historic data is currently limited by a lack of accurate taxonomic information for the majority of ancient cetacean remains. We then discuss current opportunities presented by molecular identification methods such as DNA barcoding and collagen peptide mass fingerprinting (zooarchaeology by mass spectrometry), and highlight the importance of molecular identifications in assessing ancient species’ distributions through a case study focused on the Mediterranean. We conclude by considering high-throughput molecular approaches such as hybridization capture followed by next-generation sequencing as cost-effective approaches for enhancing the ecological informativeness of these ancient sample sets.

This article is part of the themed issue ‘From DNA barcodes to biomes’.

## 1. Introduction

Humans have been exploiting cetaceans for thousands of years, first through the opportunistic use of stranded or drift whale carcasses, and subsequently by active hunting [1–4]. Their value came from the use of meat and blubber as food, blubber as fuel in oil-burning lamps, teeth (of odontocetes) as a valuable form of ivory, baleen (of mysticetes) as a raw-material source and bones used for building purposes, tool production, and as solid fuel (given their high oil content) [1,5–8]. Intensive human exploitation (particularly the industrial hunting practices of the nineteenth and early twentieth centuries), as well as other anthropogenic influences (sonar, ship strikes, habitat degradation, etc.), reduced the size of whale populations worldwide and even extirpated some local populations [9,10], including the eastern North Atlantic populations of right whales (*Eubalaena glacialis*) [11] and the Atlantic gray whale populations (*Eschrichtius robustus*) [12]. The past few decades have witnessed major efforts in the conservation of whales, including the 1984 moratorium on commercial whaling instituted by the International Whaling Commission (IWC), and the Convention on the International Trade of Endangered Species (CITES). Although many whale species are now protected and some populations are recovering [9,13], cetacean conservation is still an ecological priority for many countries. Developing informed conservation policies and sustainable management plans requires accurate historic data on cetacean abundance and distribution at various stages in their interactions with humans. Archaeological and palaeontological records are key to the reconstruction of these ecological baselines [14], but they have been dramatically underused, largely because of the challenges associated with the taxonomic identification of ancient whale bones. Molecular methods have advanced substantially in the past few decades, and molecular barcoding now provides a new opportunity to decipher and maximize the information potential of the archaeological and palaeontological records.

In this article, we review the challenges with identifying ancient whale bones, as well as the opportunities provided by new molecular identification methods. We begin by summarizing the limitations inherent to taxonomic identification based on traditional anatomical methods, illustrated with a case study from the North Sea on the proportion of unidentified archaeological cetacean remains housed in museums and repositories. We then discuss the opportunities for more accurate identifications made possible by molecular analyses, and demonstrate the need for molecular validation through a case study comparing anatomical and molecular identifications of whale bones from Mediterranean archaeological contexts. Finally, we conclude by presenting future perspectives for molecular methods, including high-throughput approaches for the study of ancient cetacean assemblages.

## 2. Limitations in identifying whale bones using anatomical methods

Despite the millennia of human–cetacean interactions, the research potentials of palaeontological and archaeozoological cetaceans have received very little attention, in large part because of the difficulty in identifying (often fragmentary) ancient whale bones to the genus or species level using comparative anatomy methods. Compared with other large mammals, whale bone is extremely friable; composed primarily of oil-filled cancellous bone, with only a thin external cortical layer, whale bone easily breaks up into non-diagnostic fragments. When a whale is exploited through active hunting or scavenging of drift carcasses, its sheer size limits the viability for humans to transport complete anatomical elements far from the beach [15]. Thus, in archaeological contexts, the larger the animal, the less bone is transported from shore to settlement, decreasing the likelihood of finding diagnostic pieces of the skeleton. A single animal can also supply more than 40 metric tons of bone [16,17], making it difficult to distinguish the number of species or individuals represented by fragmentary remains. The use of cetacean bone as raw material for combustion or tool production further fragments and modifies the bone [5]. Even when diagnostic elements are preserved, the range of morphological variation present among and within (e.g. sexual dimorphism) species can confound taxonomic identifications [18].

These identification problems are compounded by a lack of comprehensive or easily accessible skeletal reference collections, which are usually restricted to a few large national natural history museums (e.g. National History Museum, London, UK or Naturalis in Leiden, The Netherlands) [19]. Unlike most other mammalian collections, the range of morphological variation present within each species is not well represented, and is thus not well characterized in taxonomic identification atlases for a wide diversity of bones. Indeed, the challenges with storing such huge specimens mean that repositories do not typically curate more than one or two individuals from each species, often only retaining particularly diagnostic elements, such as the cranium. Collections are particularly incomplete for populations that were extirpated prior to the creation of modern museum collections (from the eighteenth century), such as the North Atlantic right whale (functionally extinct in the eastern North Atlantic [11]) or the Atlantic population of the gray whale (extinct [12]). Even the most complete collections may not serve as representative guides for ancient remains, as archaeological specimens may be considerably larger than museum specimens curated relatively recently, due to the diminution in the overall size of mature animals following the advent of modern whaling [18].

These difficulties create substantial gaps and biases in the archaeological record, with ramifications for understanding past human interactions with these marine mammals. For example, in a study of whale remains in the Western Isles (northwest of Scotland), Mulville [5] noted an increase in both the proportion and taxonomic diversity of whale bones from the later Bronze Age through to the Norse Age, with an increase in the proportion of large whale species. However, while progressively more species were identified through time, an increasing proportion of remains were not taxonomically identifiable; only 30 of 568 examined whale bones could be identified to species, largely due to extensive modification or burning of the bones [5]. Similarly, in a collection of 50 archaeological specimens from seven North Atlantic archaeological sites, ranging from the Mesolithic until the Early Modern period, most of the bone fragments could be morphologically identified only to ‘marine mammal’ or ‘cetacean’ [20]. Finally, a study in the Northeast Pacific coast of North America found that although whale bones were recovered from Nuu-chah-nulth (Nootka) sites as early as 4000 BP (before present), fewer than 20% of these could be identified to species [21].

### (a) Case study: ancient cetacean assemblages in the North Sea

**Figure 1.**
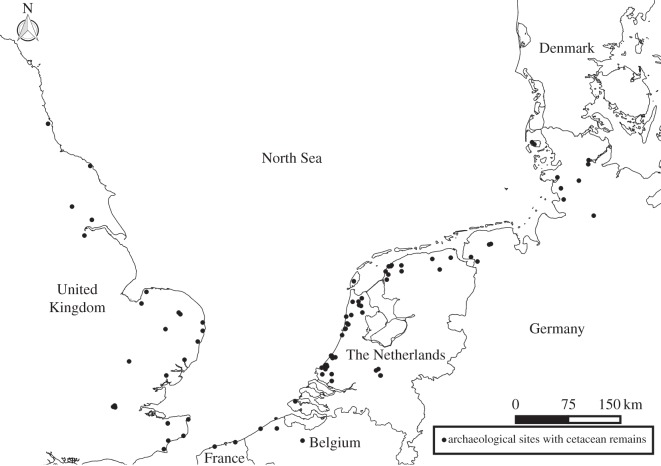
Location of southern North Sea archaeological sites with cetacean remains (including the east coast of England (*n* = 27), the French region of Nord-Pas-de-Calais (*n* = 2), Belgium (*n* = 4), The Netherlands (*n* = 56) and the North Sea coast of Germany (*n* = 13).

A case study from the southern North Sea (figure 1) illustrates the difficulties with the identification of cetacean species in the zooarchaeological record. This synthesis of faunal data from published archaeological reports revealed at least 102 sites with preserved cetacean remains, the majority of which date to the Early Medieval period [22]. Of the 616 remains recovered, less than half (*n* = 306) could be morphologically identified to the species level through traditional comparative anatomy methods (electronic supplementary table S1). Furthermore, among these taxonomically identified bones 119 originated from a single site, the Early Medieval site of Flixborough (represented by 115 common bottlenose dolphin *Tursiops truncatus*, three minke whale *Balaenoptera acutorostrata* and one killer whale *Orcinus orca*) [23].

Overall, most of the identified specimens across all 102 sites represented dolphins or porpoises. The taxonomic identification of these small species is significantly easier, as a greater proportion of entire bones are preserved and more complete reference collections are available. Only 12 specimens (less than 4% of the identified assemblage) were identified as baleen whale species. The fact that half of these baleen whales were identified as North Atlantic right whale demonstrates the significance of these ancient sample sets. Indeed, this species has all but disappeared from the North Sea, but its prevalence in these archaeological remains (albeit within an extremely small sample set) hints at its potential historic abundance within the region. It is highly likely that a significant proportion of the unidentified cetacean specimens are also from large (baleen) whales, as they are less likely to be taxonomically identified if they are in a fragmented state compared with smaller species (see discussion in [20]). Human behaviour may also preferentially increase fragmentation of large species compared with their smaller counterparts. Compared with dolphins or porpoises, baleen whales provide a more abundant supply of bone, with a thicker cortex, making them better suited as a raw material for tool production [24,25]. The lipid content of large whales is also higher than that of dolphins [26] making them more desirable as sources of biofuels. The deliberate fragmentation of these oil-rich elements to liberate the oil or maximize the surface area for burning decreases the likelihood of morphological identification [16,27]. In the North Sea, and in other regions, this lack of taxonomic precision limits our ability to detect historic changes in cetacean distribution and abundance, and document how these populations have been impacted by human activities.

## 3. Opportunities from molecular identification techniques

### (a) DNA barcoding

**Figure 2.**
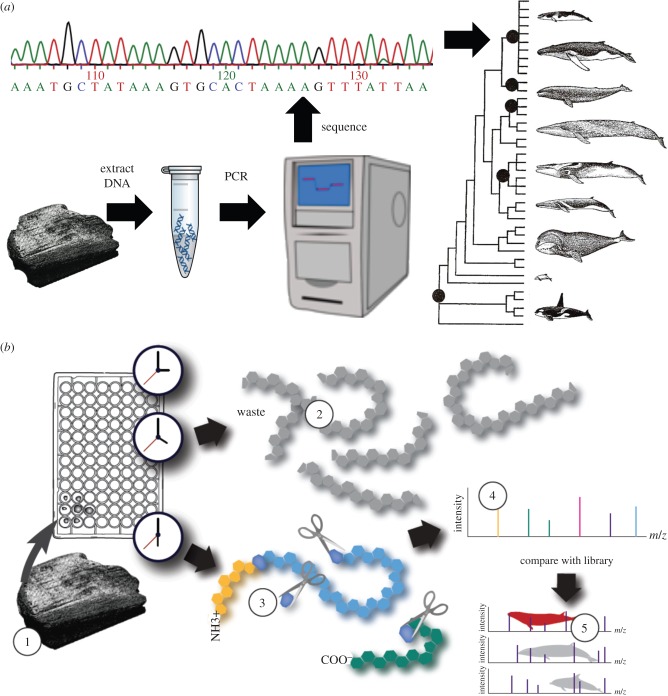
Two established methods for the molecular identification of ancient cetacean remains: **(***a*) DNA barcoding: DNA is extracted from the sample in a clean room, and PCR-amplified targeting short fragments of mtDNA. Resulting sequences are compared with a databank of known sequences for taxonomic identification. (*b*) ZooMS: samples are (1) demineralized in a weak acid solution; (2) collagen is gelatinized by heating at 65°C in an ammonium bicarbonate buffer; the collagen is then (3) enzymatically cleaved into peptides, which are spotted with a matrix onto a target plate. The masses of the peptides are measured following desorption/ionization of the sample using laser energy (MALDI) and (4) the peptide masses estimated by time of flight (TOF). The presence of specific peptides (5) is used for taxonomic identification.

Over the last two decades, molecular methods have been increasingly applied to the problem of cetacean identification, but their primary focus was the study of contemporary populations. Indeed, given the 1984 moratorium on commercial whaling by the IWC, their protected status under CITES and the many national laws protecting particular species and populations, accurate taxonomic identification of whale products has become essential to differentiate products obtained from legal versus illegal exploitation or trade. For example, identification to the species or even population level may be key to assessing whether whale products (skin, blubber, meat) sold in domestic markets have a legal origin (e.g. if they come from small odontocetes not covered by the IWC moratorium, or from populations exploited under aboriginal subsistence permits) or not. However, such products are often processed in ways that render morphological identification impossible. Considering that such processing (cooking, salting, drying, marinating) may significantly degrade DNA, early molecular studies targeted mitochondrial DNA (mtDNA), amplifying relatively short diagnostic fragments (150–500 bp) of the control region or cytochrome *b* (cyt*b*) gene to identify taxa and to estimate geographical provenience [28,29] (figure 2*a*). By comparing the resulting sequences to a databank of known species and populations, it is possible to evaluate the relationships between known and unknown samples by parsimony or maximum-likelihood criteria, with the reliability of phylogenetic relationships analysed by bootstrapping procedures [30]. Tree-based approaches, however, can be problematic in situations where relationships among interbreeding organisms are not hierarchical, or where species are polyphyletic [31]. In these cases, taxonomic identifications may be more robust when sequences are analysed using vector- or distance-based clustering methods to evaluate distribution of derived character states [31,32].

In the 1990s, genetic databases were limited in the number of type species and populations represented, and thus taxonomic identifications and/or phylogeographic analyses were often tentative [29]. Over the last two decades, follow-on studies in cetacean systematics and phylogeography [33,34], the development of comprehensive wildlife DNA registers [35] and validated reference sequence databanks (e.g. DNA surveillance [36]) have significantly enhanced the ability to ‘barcode’ morphologically ambiguous cetacean remains, not only in markets but also animals caught as fisheries bycatch or derived from strandings. Beyond species identification, mtDNA and nuclear DNA (short tandem repeats (STRs), actin sequences) are being applied to quantify the minimum number of individuals entering trade [37], estimate the total catches resulting from market meat [38] or even to track the life history of an individual whale [39].

Studies have also explored the potential for applying these molecular methods to palaeontological or archaeological remains. As with modern whale product identification, studies of ancient specimens primarily concentrated on recovering short diagnostic fragments of mtDNA control region [40,41], cyt*b* gene [42] or both [43,44] for accurate taxonomic identification. In addition to archaeological bone, DNA analysis has been applied to other whale products, such as museum samples of baleen [45,46], whale ivory or scrimshaw [40], with relative success. Although there has been the occasional large-scale study identifying hundreds of samples [47], DNA-based studies have been primarily applied to demonstrate the feasibility of these molecular techniques, or at the site level, to identify the range of species exploited within a geographically restricted region. Among the aforementioned specimens in the North Sea case study, only two have been identified through ancient DNA analysis (two fin whale specimens, at Barreau Saint Georges, France) [48]. There is thus much unexploited potential for the application of these methods to the analysis of ancient specimens. However, the relatively high cost of these analyses and the fact that they need to be done in specialized laboratories (to prevent DNA contamination) remains a limiting factor to their large-scale application.

### (b) Zooarchaeology by mass spectrometry: collagen peptide mass fingerprinting

Peptide mass fingerprinting (PMF) has been widely used as a rapid and cost-effective protein identification method based upon the pattern of mass-to-charge (*m*/*z*) ratios [49]. Most recently, it has been developed for the most abundant protein in archaeological bone: collagen (figure 2*b*). In mammals, collagen is composed of two alpha 1 chains, and a third, more rapidly evolving alpha 2 chain. In collagen PMF approaches, collagen is extracted from archaeological bone, followed by enzymatic digestion, which cleaves proteins at specific amino acid sites producing a characteristic mixture of peptides. The peptides are analysed through matrix-assisted laser desorption/ionization mass spectrometry (MALDI-MS), producing a ‘peptide mass fingerprint’ based on their respective *m*/*z* ratios. Species identification of archaeological bones can thus be accomplished by comparing collagen peptide fingerprints with the fingerprints from known samples—i.e. zooarchaeology by mass spectrometry (ZooMS) [50,51]. Collagen's relatively slow rate of evolution means that it is variable enough to discriminate between mammal genera, but is sufficiently similar to map differences across broad taxonomic groups, such as cetaceans [52,53]. The ZooMS approach has been developed and tested on North Atlantic cetacean species, providing a rapid and cost-effective identification screening approach often to the genus or species level [20,54].

The advantages of a collagen versus DNA-based approach for identifying ancient samples are numerous. First, collagen is a remarkably robust protein, and recent evidence suggests that collagen survives at least 10 times longer than DNA, preserving even in tropical climates where DNA preservation is poor [53,55]. Unlike PCR-based approaches, which can be limited by primer specificity, ZooMS can be applied to highly fragmented non-diagnostic bone without any prior taxonomic knowledge [56]. As ZooMS does not require the amplification of degraded ancient molecules, the risk of false positives from contaminating modern template or previously amplified PCR products is also reduced. Moreover, collagen can be recovered and analysed using a non-destructive ammonium bicarbonate buffer, which enables bone samples or artefacts to be analysed without destructive sampling [57]. ZooMS, however, does have its limitations: due to the relatively slow mutation rate of collagen, taxonomic precision is often limited to the genus level. For example, although most baleen whale species can be distinguished, ZooMS cannot currently differentiate between bowhead and right whale, or among some dolphin species [20]. Additionally, robust identifications often require the successful recovery of multiple diagnostic peptides. Thus, mass spectra from poorly preserved samples may only allow identification to higher taxonomic levels (family, order) if diagnostic peptide markers are absent. Genetic methods may be required to clarify species identity and are certainly required for identification to the subspecies or population levels. However, applying ZooMS as an initial screening method can provide a cost-effective preliminary identification, as well as insight into overall biomolecular preservation and the likely success for subsequent DNA analysis [58] or radiocarbon dating [59].

Biomolecular identification approaches such as DNA barcoding and ZooMS can offer robust taxonomic identifications of ancient cetaceans, however, they can be limited by taphonomic histories and biomolecular preservation. Some studies, for example, have noted a high presence of inhibitory substances in ancient whale bones, compromising the success of PCR amplifications [60–62]. Also, archaeological whale bone has often been burned, limiting the quantity and quality of DNA and collagen that can be obtained from the samples [63,64]. Likewise, biomolecular degradation can be extensive in samples recovered from tropical or sub-tropical environments [65,66]. Despite these challenges, biomolecular identifications of archaeological cetaceans can be applied to many specimens that remain currently unidentified, and thus make a decisive contribution to reconstructing the ecology and population history of cetacean species.

### (c) Importance of molecular identifications: a case study validating ancient cetacean specimens in the Mediterranean Sea

**Figure 3.**
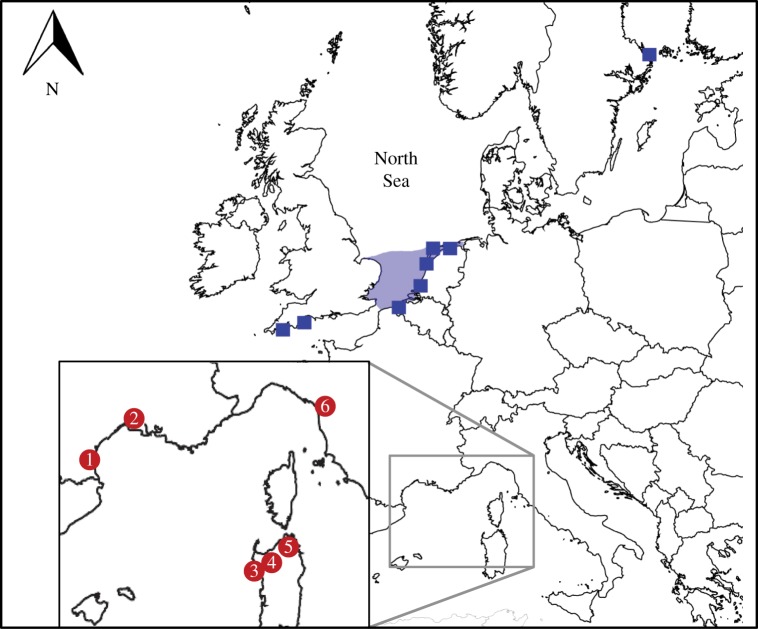
Map displaying locations of confirmed [68] palaeontological gray whale finds in the northeast Atlantic (filled squares, and shaded area representing southern bight of the North Sea) and the locations of the Mediterranean archaeological sites tested here (circled numbers: (1) Saint Sauveur; (2) Cougourlude and Saint Martin; (3) Villa Sant'Imbenia; (4) Porto Torres; (5) Nuraghe Lu Brandali and (6) San Rocchino.

In order to illustrate the necessity of validating osteological identifications of cetaceans, we present a case study of 17 pre-industrial cetacean specimens from six sites around the Mediterranean Sea (table 1, figure 3; electronic supplementary material, table S2). This collection is particularly meaningful as it includes five specimens previously identified through comparative anatomy methods as Atlantic gray whale (*E. robustus*) [67]. The gray whale is currently found only in the North Pacific, and the circumstances of its disappearance from the North Atlantic remain a mystery [12] as this population left very few historical, archaeological or palaeontological traces. Fewer than 60 remains are known from both sides of the Atlantic, and the 34 records in the eastern North Atlantic (dated from the Late Pleistocene to the eighteenth century), are nearly all from the North Sea [68] (figure 3). The restricted spatial distribution of these bones is probably a poor reflection of their actual past range; indeed, habitat modelling predicts gray whales would have also occurred further south, including the Bay of Biscay, and to a lesser extent, the Mediterranean Sea [68]. Owing to this paucity of remains, the reliability of each new gray whale identification outside the currently known distribution is potentially crucial to our understanding of the distribution and ecology of this population. Twelve additional Mediterranean samples (identified only to the level of cetacea) were also included in this study to further increase the possibility of detecting gray-whale remains.

**Table 1.** mtDNA taxonomic identifications of Mediterranean archaeological cetacean bones. Samples listed in bold indicate those previously identified as gray whale remains through anatomical methods [67]; additional detail provided in electronic supplementary material, table S2. The identified species are right whale (*Eubalaena glacialis*), fin whale (*Balaenoptera physalus*), sperm whale (*Physeter catodon*) and Cuvier's beaked whale (*Ziphius cavirostris*).

laboratory code	archaeological site	chronology	DNA species ID	collagen PMF (ZooMS) ID
WH501	Saint Martin, s. France	Late Antiquity	right whale	right/bowhead whale
WH502	Cougourlude, s. France	Roman	no amplification	no ID
WH503	Cougourlude, s. France	Roman	fin whale	no ID
WH504	Cougourlude, s. France	Roman	no amplification	no ID
**WH505**	**Saint Sauveur, s. France**	Roman	**fin whale**	**fin whale**
WH506	Saint Sauveur, s. France	Iron Age	fin whale	fin whale
**WH507**	**Saint Sauveur, s. France**	Iron Age	**fin whale**	**fin whale**
**WH508**	**Saint Sauveur, s. France**	Iron Age	**sperm whale**	**sperm whale**
**WH509**	**Saint Sauveur, s. France**	Iron Age	**no amplification**	**baleen whale (Mysticeti)**
**WH510**	**Saint Sauveur, s. France**	Late Antiquity	**fin whale**	**fin whale**
WH511	Saint Sauveur, s. France	Iron Age	fin whale	fin whale
WH512	Saint Sauveur, s. France	Iron Age	fin whale	fin whale
WH513	Saint Sauveur, s. France	Iron Age	fin whale	fin whale
WH801	Nuraghe Lu Brandali, Sardinia	Bronze Age	Cuvier's beaked whale	beaked whale
WH802	Porto Torres, Sardinia	Roman	no amplification	fin whale
WH803	Villa Sant'Imbenia, Sardinia	Early Middle Age	fin whale	fin whale
WH804	San Rocchino, Tuscany, Italy	Iron Age	no amplification	fin whale

We analysed the 17 ancient bones using DNA barcoding (cyt*b* mtDNA analysis) and ZooMS (methods described in the electronic supplementary material), identifying 11 fin whale (*Balaenoptera physalus*), one sperm whale (*Physeter catodon*), one right whale (*E. glacialis*) and one Cuvier's beaked whale (*Ziphius cavirostris*). The relative merits of both techniques in terms of their precision and robusticity are exemplified here. For example, although ZooMS identified two samples only as ‘beaked whale’ and ‘bowhead/right whale’, respectively, DNA provided the resolution to confirm these as Cuvier's beaked whale and right whale. Although ZooMS may be less precise, it may be more successful with poorly preserved samples: three samples that failed DNA analysis were identified through ZooMS as two fin whales and one baleen whale (Mysticeti), respectively. For the latter sample, a higher taxonomic resolution was not possible due to a lack of high molecular–weight diagnostic peptide markers in the mass spectra (electronic supplementary material, figure S1). Despite the advantages of applying both techniques to the same assemblage, two samples failed to produce identification using either molecular method, illustrating the limitations of working with degraded archaeological materials.

Of the five samples previously believed to correspond to gray whale, three were identified as fin whale and one as sperm whale, with the fifth identified only as a ‘baleen whale’. Thus, none of the samples could be confirmed as gray whale using molecular approaches. These results illustrate the necessity of validating anatomical identifications using molecular techniques, even more so as this is not the first study to reveal previously misidentified whale remains. For example, a mtDNA-based analysis of seventeenth century Basque whaling remains determined that morphological identifications previously assigned to right whale were in fact bowhead whale [69]. Likewise, molecular analyses of archaeological cetacean fragments from Tierra del Fuego believed to correspond to the remains of a single animal within a hunter–gatherer midden revealed the presence of multiple whale species as well as non-cetaceans (e.g. human, pinniped) [42]. This latter study also demonstrated that available museum reference specimens may themselves have been incorrectly identified using anatomical methods. These and other studies (e.g. [47,70]) collectively highlight the crucial need for the molecular screening of existing and future zooarchaeological collections containing whale bones.

The hypothesis that gray whales previously migrated to calving grounds in the Mediterranean Sea was largely supported by the presence of these five ‘putative’ gray whale bones [66]—identifications which failed to be confirmed by molecular methods in this study. However, records for fin, sperm and beaked whales are in agreement with the composition of the extant Mediterranean whale assemblage: fin whales are the most common species in the Mediterranean, with highest abundance in the Corso-Ligurian basin and Gulf of Lyon; and sperm and Cuvier's beaked whales are also regular species in the Mediterranean Sea, although less common than fin whale [71]. By contrast, the right whale specimen indicates a possible change in the regional whale composition. Indeed, not only is this species currently absent from the Mediterranean, it is also extremely rare in the historical record, with only three known sightings (Italy 1877, Alger 1888 and Sardinia 1991) [72]. In the archaeological record, there is indirect proof of its prior presence at the entrance of Gibraltar: several plates of two barnacle species specific to right whales found in the Upper Magdalenian layers of a cave in Málaga, Southern Spain [73]. The bone specimen identified in this study is thus the first direct archaeological evidence of right whale in the Mediterranean Sea. Given that the likelihood of vagrant individuals ending up in the archaeological record is small, this result (combined with the Málaga study) suggests that this species may have been regularly present in the Mediterranean before its near-extirpation from the eastern North Atlantic. Our results illustrate the importance of the zooarchaeological record for understanding the past distribution, abundance and ecology of whales.

## 4. Future perspectives: high-throughput methods

The need for accurate molecular identifications, coupled with the large proportion of unidentified archaeological and palaeontological remains, emphasizes the importance of high-throughput methods in future cetacean barcoding projects. Traditional mtDNA barcoding approaches are well established, and typically provide robust species identifications (with the exception of cross-species hybrids [39]) for modern, degraded and ancient samples. However, the need for careful sample preparation, clean-room extraction and replicability when working with ancient remains can significantly increase the laboratory time and associated costs when working with many hundreds of remains. ZooMS, on the other hand, can more easily be scaled up for large datasets: using a plate approach, up to 96 samples can be processed at one time [74], potentially allowing for up to 1000 samples to be analysed per week [75]. Although ZooMS is a cost-effective, high-throughput screening method for large sample sets, it often lacks the taxonomic precision offered by genetic analysis. Given the importance of accurate molecular identifications for ancient whale bones and the large proportion of unidentified archaeological and palaeontological remains, the future will probably rely on next-generation sequencing (NGS) approaches, which can offer both taxonomic precision and bulk processing. Here, we review the advantages and limitations of NGS methods, including hybridization capture approaches, and their application to modern and ancient ecological studies.

### (a) Next-generation sequencing methods

**Figure 4.**
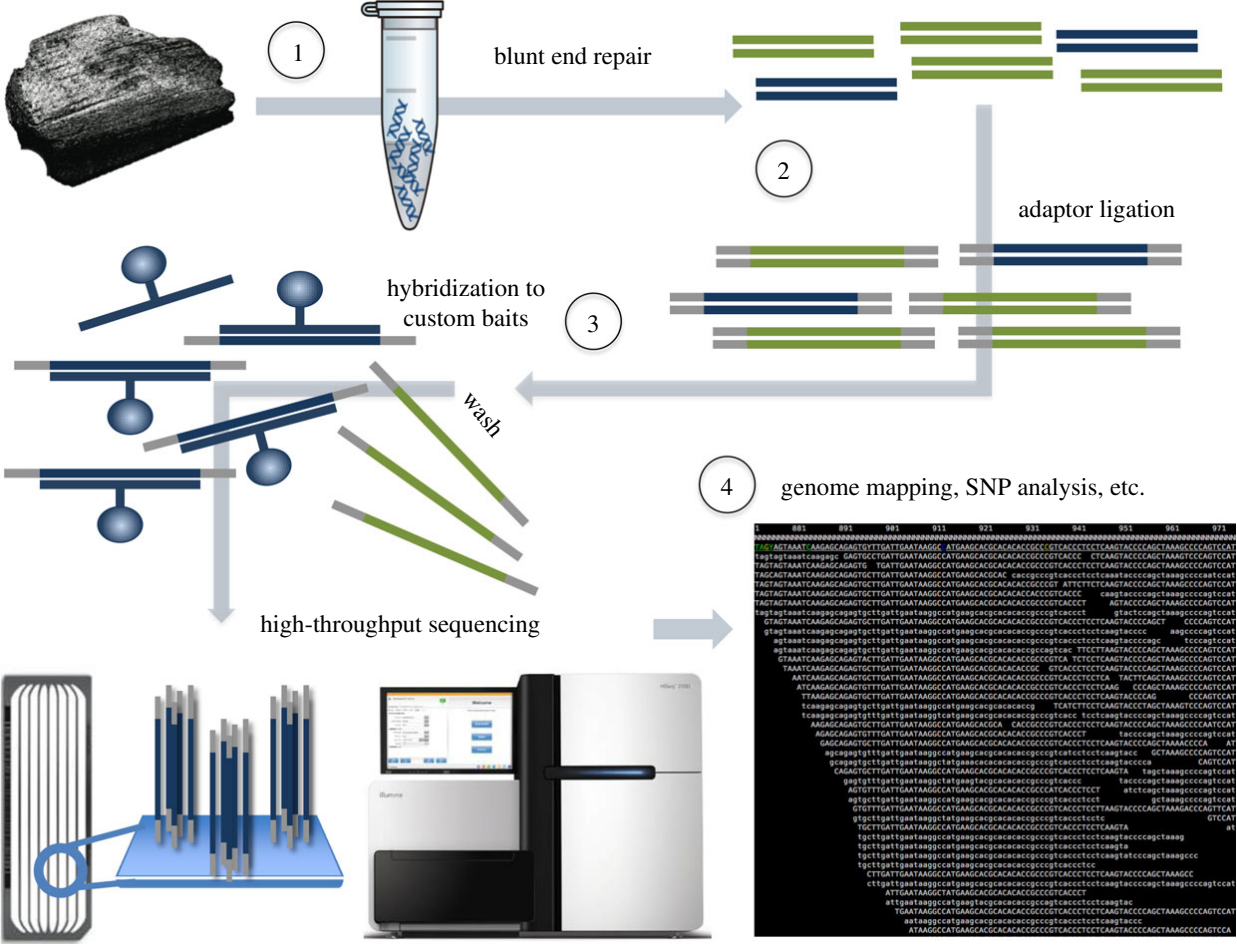
DNA hybridization capture coupled to NGS: (1) DNA is extracted in a clean room and (2) NGS libraries are built from the extract; (3) the libraries are enriched for specific DNA sequences by hybridization to custom designed baits, and non-target templates are washed away; (4) enriched libraries are sequenced on a NGS platform, and the resulting data are analysed bioinformatically.

The advent of high-throughput or NGS methods has revolutionized the application of ancient genetics, massively enhancing the ability to recover ancient DNA templates from degraded remains. Although whole-genome ‘shotgun’ approaches have been attempted for species identification (most notably to refine the systematics of ancient hominids [76,77]), this approach is limited by the generally low percentage of endogenous DNA in ancient remains and the lack of nuclear reference genomes in public databases like GenBank or Ensembl [78]. Until comprehensive genome databases are available, mitochondrial genes and genomes and informative nuclear genes will primarily be the markers of choice for ancient cetacean identification, with DNA target enrichment followed by NGS as the most feasible high-throughput method for data acquisition [79]. Enrichment (or capture) methodologies immobilize the target DNA regions through hybridization to single-stranded DNA or RNA probes with high sequence homology (figure 4). Following DNA extraction and library preparation, custom probes are used to immobilize the target DNA either on a solid phase (e.g. surface of a microarray) or in-solution using biotinylated baits. Non-homologous DNA templates are then washed away, the target DNA is eluted off the probes and sequenced using NGS methods. Hybridization capture of entire mitochondrial genomes (mitogenomes) has become increasingly common for ancient or degraded DNA studies [80–82], as it allows targeting of DNA even from highly degraded samples, and can be scaled up for population-level analyses.

Enrichment approaches are particularly useful for increasingly old samples, or those from tropical climates where preserved DNA templates may be degraded beyond the fragment length feasible for traditional PCR amplicons [83,84]. Moreover, complete mitogenomes have been shown to provide more robust topologies and estimates of divergence times than shorter mitochondrial sequences [85,86]. While NGS approaches are still considerably more expensive than capillary sequencing when dealing with small numbers of samples, they can be far more cost-effective if designed in a way that minimizes unusable sequences (e.g. environmental contamination, non-target DNA) and captures information for the maximal number of samples [87]. Hybridization probes can be designed to capture and simultaneously sequence up to 100 specimens on a single lane of NGS instrumentation significantly reducing the per-sample costs, and providing mitogenome data for both initial species identification and subsequent phylogenetic analyses. Although the hybridization probes can be specifically designed to capture mitogenomes from single or multiple cetacean species, recent studies have demonstrated that ‘generic’ probes are capable of recovering mitogenomes from even phylogenetically distinct taxa [88–90]. Furthermore, palaeontological studies have already demonstrated the advantages of pairing ZooMS with NGS methods, by first screening thousands of bone fragments using ZooMS, followed by mitogenome capture of particular species of interest [75].

### (b) Potential contributions of next-generation sequencing to cetacean ecology

NGS approaches are beginning to be applied more routinely to modern cetacean populations, recovering full mitogenomes [91–95], genomic single nucleotide polymorphisms (SNPs) [96,97] or even complete nuclear genomes [98] to develop more nuanced models of evolutionary systematics and population histories for various cetacean species. To date, hybridization capture has not yet been extensively applied to ancient marine species. The capture of ancient Steller's sea cow nuclear genes [99], and ancient killer whale mitogenomes [100], however, demonstrate the utility of this approach for revealing both broad interordinal evolutionary systematics as well as more recent radiations. Molecular analyses targeting only fragments of mtDNA in palaeontological and archaeological remains have already shed light on the past distribution and abundance of cetaceans over thousands of years, and the extent to which these populations have been impacted by humans [101]. Examples include recent studies on the gray whale (*E. robustus*) [102], bowhead whale (*Balaena mysticetus*) [103,104], North Atlantic right whale (*E. glacialis*) [46] and Hector's dolphin (*Cephalorhynchus hectori*) [105], which have provided more accurate estimates of cetacean genetic diversity and population sizes prior to their overexploitation. These data provide crucial baselines for conservation and management efforts, for example, as part of IWC's mandate to allow whale populations to recover to sustainable levels. Integrated with long-term climatic data and predictive habitat modelling, they can shed light onto how populations will respond to future anthropogenic change [68,70].

## 5. Conclusion

Molecular methods are already proving crucial to our understanding of the past distribution and abundance of whale species and much scope remains to expand their application to existing zooarchaeological collections. With further refinement of these methods and the augmentation of cetacean genomic reference datasets, we will be able to obtain increasingly fine-grained identifications to the subspecies, ecotype and population levels. The systematic integration of well-dated archaeological and palaeontological remains with high-throughput molecular analysis methods will reveal changes in habitat, genetic diversity and population abundance associated with climatic and anthropogenic factors through millennial timescales [101,106].

## Supplementary Material

Supplementary information

## Data accessibility

DNA sequences: GenBank accessions KT923090–KT923101.

## References

[1] Clark G 1947 Whales as an economic factor in prehistoric Europe. Antiquity 21, 84–104. (10.1017/S0003598X00016513)

[2] Monks G (ed.). 2005 The exploitation and cultural importance of sea mammals. Oxford, UK: Oxbow Books.

[3] Piana E 2005 Cetaceans and human beings at the uttermost part of America: a longlasting relationship in Tierra del Fuego. In The exploitation and cultural importance of sea mammals (ed. Monks G), pp. 121–137. Oxford, UK: Oxbow Books.

[4] Kandel AW, Conard NJ 2003 Scavenging and processing of whale meat and blubber by Later Stone Age people of the Geelbek Dunes, Western Cape Province, South Africa. S. Afr. Archaeol. Bull. 58, 91–93. (10.2307/3889306)

[5] Mulville J 2002 The role of cetacea in prehistoric and historic Atlantic Scotland. Int. J. Osteoarchaeol. 12, 34–48. (10.1002/oa.611)

[6] Dawson PC 2001 Interpreting variability in Thule Inuit Architecture: a case study from the Canadian High Arctic. Am. Antiq. 66, 453–470. (10.2307/2694244)

[7] Monks GG, McMillan AD, Claire DES 2001 Nuu-Chah-Nulth whaling: archaeological insights into Antiquity, species preferences, and cultural importance. Arct. Anthropol. 38, 60–81.

[8] Pétillon J-M 2008 First evidence of a whale-bone industry in the western European Upper Paleolithic: Magdalenian artifacts from Isturitz (Pyrénées-Atlantiques, France). J. Hum. Evol. 54, 720–726. (10.1016/j.jhevol.2007.12.006) 18272202

[9] Clapham PJ, Young SB, Brownell RL 1999 Baleen whales: conservation issues and the status of the most endangered populations. Mamm. Rev. 29, 37–62. (10.1046/j.1365-2907.1999.00035.x)

[10] Scott Baker C, Clapham PJ 2004 Modelling the past and future of whales and whaling. Trends Ecol. Evol. 19, 365–371. (10.1016/j.tree.2004.05.005) 16701287

[11] Reeves RR, Smith TD, Josephson EA 2007 Near-annihilation of a species: right whaling in the North Atlantic. In The urban whale: North Atlantic right whales at the crossroads (eds Kraus SD, Rolland RM), pp. 39–74. Cambridge, MA: Harvard University Press.

[12] Lindquist O 2000 The North Atlantic gray whale (Eschrichtius robustus): an historical outline based on Icelandic, Danish-Icelandic, English and Swedish sources dating from ca 1000 AD to 1792. St. Andrews, UK: University of St. Andrews.

[13] Alter SE, Rynes E, Palumbi SR 2007 DNA evidence for historic population size and past ecosystem impacts of gray whales. Proc. Natl Acad. Sci. USA 104, 15 162–15 167. (10.1073/pnas.0706056104) 17848511 PMC1975855

[14] Lyman RL 2006 Paleozoology in the service of conservation biology. Evol. Anthropol. 15, 11–19. (10.1002/evan.20083)

[15] Smith AB, Kinahan J 1984 The invisible whale. World Archaeol. 16, 89–97. (10.1080/00438243.1984.9979918)

[16] Monks GG 2005 An oil utility index for whale bones. In The exploitation and cultural importance of sea mammals (ed. Monks GG), pp. 138–153. Oxford, UK: Oxbow Books.

[17] Savelle JM 1997 The role of architectural utility in the formation of zooarchaeological whale bone assemblages. J. Archaeol. Sci. 24, 869–885. (10.1006/jasc.1996.0167)

[18] Szabo VE 2008 Monstrous fishes and the mead-dark sea: whaling in the medieval North Atlantic. Leiden, The Netherlands: Brill.

[19] Sabin RC 2005 From the Palaeolithic to the present-day: the research value of marine mammal remains from archaeological contexts and the uses of contemporary museum reference collections. In The exploitation and cultural importance of sea mammals (ed. Monks GG), pp. 1–5. Oxford, UK: Oxbow Books.

[20] Buckley M, Fraser S, Herman J, Melton ND, Mulville J, Pálsdóttir AH 2014 Species identification of archaeological marine mammals using collagen fingerprinting. J. Archaeol. Sci. 41, 631–641. (10.1016/j.jas.2013.08.021)

[21] Huelsbeck DR 1988 Whaling in the precontact economy of the central northwest coast. Arct. Anthropol. 25, 1–15.

[22] van den Hurk Y 2014 A whale of a question: history and social implications of cetacean exploitation in the North Sea area through space and time. MSc Thesis, University of Nottingham, Nottingham, UK.

[23] Dobney KM, Jaques D, Barrett J, Johnstone C 2007 Farmers, monks and aristocrats: the environmental archaeology of Anglo-Saxon Flixborough. Oxford, UK: Oxbow Books.

[24] Pétillon J-M 2013 Circulation of whale-bone artifacts in the northern Pyrenees during the late Upper Paleolithic. J. Hum. Evol. 65, 525–543. (10.1016/j.jhevol.2013.06.006) 24103459

[25] Betts MW 2007 The Mackenzie Inuit Whale Bone Industry: raw material, tool manufacture, scheduling, and trade. Arctic 60, 129–144.

[26] Higgs ND, Little CTS, Glover AG 2011 Bones as biofuel: a review of whale bone composition with implications for deep-sea biology and palaeoanthropology. Proc. R. Soc. B 278, 9–17. (10.1098/rspb.2010.1267) PMC2992730 20702457

[27] de Castilho PV 2008 Utilization of cetaceans in shell mounds from the southern coast of Brazil. Quat. Int. 180, 107–114. (10.1016/j.quaint.2007.09.015)

[28] Baker CS, Cipriano F, Palumbi SR 1996 Molecular genetic identification of whale and dolphin products from commercial markets in Korea and Japan. Mol. Ecol. 5, 671–685. (10.1111/j.1365-294X.1996.tb00362.x)

[29] Baker CS, Palumbi SR 1994 Which whales are hunted? A molecular genetic approach to monitoring whaling. Science 265, 1538–1539. (10.1126/science.265.5178.1538) 17801528

[30] Palumbi SR, Cipriano F 1998 Species identification using genetic tools: the value of nuclear and mitochondrial gene sequences in whale conservation. J. Hered. 89, 459–464. (10.1093/jhered/89.5.459) 9768497

[31] Goldstein PZ, DeSalle R 2011 Integrating DNA barcode data and taxonomic practice: determination, discovery, and description. Bioessays 33, 135–147. (10.1002/bies.201000036) 21184470

[32] DeSalle R, Egan MG, Siddall M 2005 The unholy trinity: taxonomy, species delimitation and DNA barcoding. Phil. Trans. R. Soc. B 360, 1905–1916. (10.1098/rstb.2005.1722) 16214748 PMC1609226

[33] Dalebout ML, Baker CS, Mead JG, Cockcroft VG, Yamada TK 2004 A comprehensive and validated molecular taxonomy of beaked whales, family Ziphiidae. J. Hered. 95, 459–473. (10.1093/jhered/esh054) 15475391

[34] Valsecchi E, Amos W 1996 Microsatellite markers for the study of cetacean populations. Mol. Ecol. 5, 151–156. (10.1111/j.1365-294X.1996.tb00301.x) 9147690

[35] Palsbøll PJ, Bérubé M, Skaug HJ, Raymakers C 2006 DNA registers of legally obtained wildlife and derived products as means to identify illegal takes. Conserv. Biol. 20, 1284–1293. (10.1111/j.1523-1739.2006.00429.x) 16922244

[36] Baker CS, Dalebout ML, Lavery S, Ross HA 2003 DNA-surveillance: applied molecular taxonomy for species conservation and discovery. Trends Ecol. Evol. 18, 271–272. (10.1016/S0169-5347(03)00101-0)

[37] Dalebout ML, Lento GM, Cipriano F, Funahashi N, Baker CS 2002 How many protected minke whales are sold in Japan and Korea? A census by microsatellite DNA profiling. Anim. Conserv. 5, 143–152. (10.1017/S1367943002002202)

[38] Baker CS, Cooke JG, Lavery S, Dalebout ML, Ma Y-U, Funahashi N, Carraher C, Brownell RL Jr 2007 Estimating the number of whales entering trade using DNA profiling and capture-recapture analysis of market products. Mol. Ecol. 16, 2617–2626. (10.1111/j.1365-294X.2007.03317.x) 17594434

[39] Cipriano F, Palumbi SR 1999 Genetic tracking of a protected whale. Nature 397, 307–308. (10.1038/16823)

[40] Pichler FB, Dalebout ML, Baker CS 2001 Nondestructive DNA extraction from sperm whale teeth and scrimshaw. Mol. Ecol. Notes 1, 106–109. (10.1046/j.1471-8278.2001.00027.x)

[41] Pimper LE, Remis MI, Natalie R, Goodall P, Baker CS 2009 Teeth and bones as sources of DNA for genetic diversity and sex identification of commerson's dolphins (*Cephalorhynchus commersonii*) from Tierra del Fuego, Argentina. Aquat. Mamm. 35, 330–333. (10.1578/AM.35.3.2009.330)

[42] Evans S et al. 2015 Using combined biomolecular methods to explore whale exploitation and social aggregation in hunter-gatherer-fisher society in Tierra del Fuego. J. Archaeol. Sci. Rep. 6, 757–767. (10.1016/j.jasrep.2015.10.025)

[43] Yang DY, Speller CF 2006 Co-amplification of cytochrome b and D-loop mtDNA fragments for the identification of degraded DNA samples. Mol. Ecol. Notes 6, 605–608. (10.1111/j.1471-8286.2006.01370.x)

[44] Losey RJ, Yang DY 2007 Opportunistic whale hunting on the Southern Northwest Coast: ancient DNA, artifact, and ethnographic evidence. Am. Antiq. 72, 657–676. (10.2307/25470439)

[45] Sinding M-HS, Gilbert MTP, Grønnow B, Gulløv HC, Toft PA, Foote AD 2012 Minimally destructive DNA extraction from archaeological artefacts made from whale baleen. J. Archaeol. Sci. 39, 3750–3753. (10.1016/j.jas.2012.06.020)

[46] Rosenbaum HC et al. 2000 Utility of north atlantic right whale museum specimens for assessing changes in genetic diversity. Conserv. Biol. 14, 1837–1842. (10.1046/j.1523-1739.2000.99310.x) 35701919

[47] McLeod BA, Brown MW, Moore MJ, Stevens W, Barkham SH, Barkham M, White BN 2008 Bowhead whales, and not right whales, were the primary target of 16th-to 17th-Century Basque Whalers in the Western North Atlantic. Arctic 61, 61–75.

[48] UBAAL (Unité de Bioarchéologie Animalde de Lille). 2014 Exploitation du Rorqual commun au début de Xie s. près de Dunkerque. See http://perso.univ-lille3.fr/~toueslati/spip.php?breve5&lang=fr (accessed 18 August 2014).

[49] Webster J, Oxley D 2009 Protein identification by peptide mass fingerprinting using MALDI-TOF mass spectrometry. In The protein protocols handbook (ed. Walker JM), pp. 1117–1129. Totowa, NJ: Humana Press.

[50] Buckley M, Collins M, Thomas-Oates J, Wilson JC 2009 Species identification by analysis of bone collagen using matrix-assisted laser desorption/ionisation time-of-flight mass spectrometry. Rapid Commun. Mass Spectrom. 23, 3843–3854. (10.1002/rcm.4316) 19899187

[51] Collins M, Buckley M, Grundy HH, Thomas-Oates J, Wilson J, van Doorn N 2010 ZooMS: the collagen barcode and fingerprints. Spectroscopy Europe 22, 6.

[52] Buckley M 2015 Ancient collagen reveals evolutionary history of the endemic South American ‘ungulates’. Proc. R. Soc. B 282, 20142671 (10.1098/rspb.2014.2671) PMC4426609 25833851

[53] Welker F et al. 2015 Ancient proteins resolve the evolutionary history of Darwin's South American ungulates. Nature 522, 81–84. (10.1038/nature14249) 25799987

[54] Kirby DP, Buckley M, Promise E, Trauger SA, Holdcraft TR 2013 Identification of collagen-based materials in cultural heritage. Analyst 138, 4849–4858. (10.1039/c3an00925d) 23807214

[55] Buckley M, Whitcher Kansa S, Howard S, Campbell S, Thomas-Oates J, Collins M 2010 Distinguishing between archaeological sheep and goat bones using a single collagen peptide. J. Archaeol. Sci. 37, 13–20. (10.1016/j.jas.2009.08.020)

[56] Welker F, Soressi M, Rendu W, Hublin J-J, Collins M 2015 Using ZooMS to identify fragmentary bone from the Late Middle/Early Upper Palaeolithic sequence of Les Cottés, France. J. Archaeol. Sci. 54, 279–286. (10.1016/j.jas.2014.12.010)

[57] van Doorn NL, Hollund H, Collins MJ 2011 A novel and non-destructive approach for ZooMS analysis: ammonium bicarbonate buffer extraction. Archaeol. Anthropol. Sci. 3, 281–289. (10.1007/s12520-011-0067-y)

[58] von Holstein ICC et al. 2014 Searching for Scandinavians in pre-Viking Scotland: molecular fingerprinting of Early Medieval combs. J. Archaeol. Sci. 41, 1–6. (10.1016/j.jas.2013.07.026)

[59] Harvey VL, Egerton VM, Chamberlain AT, Manning PL, Buckley M 2016 Collagen fingerprinting: a new screening technique for radiocarbon dating ancient bone. PLoS ONE 11, e0150650 (10.1371/journal.pone.0150650) 26938469 PMC4777535

[60] Tebbutt SJ, Stewart RE, Hill DF 2000 Isolation and characterisation of DNA from whale bone. J. R. Soc. NZ 30, 365–371. (10.1080/03014223.2000.9517628)

[61] Morin PA, Mccarthy M 2007 Highly accurate SNP genotyping from historical and low-quality samples. Mol. Ecol. Notes 7, 937–946. (10.1111/j.1471-8286.2007.01804.x)

[62] Arndt UM 2011 Ancient DNA analysis of northeast Pacific humpback whale (*Megaptera novaeangliae*). PhD Thesis, Simon Fraser University, Burnaby, Canada.

[63] Collins MJ et al. 2002 The survival of organic matter in bone: a review. Archaeometry 44, 383–394. (10.1111/1475-4754.t01-1-00071)

[64] Ottoni C, Koon HEC, Collins MJ, Penkman KEH, Rickards O, Craig OE 2009 Preservation of ancient DNA in thermally damaged archaeological bone. Naturwissenschaften 96, 267–278. (10.1007/s00114-008-0478-5) 19043689

[65] Allentoft ME et al. 2012 The half-life of DNA in bone: measuring decay kinetics in 158 dated fossils. Proc. R. Soc. B. 279, 4724–4733. (10.1098/rspb.2012.1745) PMC3497090 23055061

[66] Hofreiter M, Paijmans JLA, Goodchild H, Speller CF, Barlow A, Fortes GG, Thomas JA, Ludwig A, Collins MJ 2015 The future of ancient DNA: technical advances and conceptual shifts. Bioessays 37, 284–293. (10.1002/bies.201400160) 25413709

[67] Macé M 2003 Did the Gray whale, *Eschrichtius robustus*, calve in the Mediterranean? Lattara 16, 153–164.

[68] Alter SE et al. 2015 Climate impacts on transocean dispersal and habitat in gray whales from the Pleistocene to 2100. Mol. Ecol. 24, 1510–1522. (10.1111/mec.13121) 25753251

[69] Rastogi T, Brown MW, McLeod BA, Frasier TR, Grenier R, Cumbaa SL, Nadarajah J, White BN 2004 Genetic analysis of 16th-century whale bones prompts a revision of the impact of Basque whaling on right and bowhead whales in the western North Atlantic. Can. J. Zool. 82, 1647–1654. (10.1139/z04-146)

[70] Foote AD et al. 2013 Ancient DNA reveals that bowhead whale lineages survived Late Pleistocene climate change and habitat shifts. Nat. Commun. 4, 1677 (10.1038/ncomms2714) 23575681

[71] Reeves RR, Notarbartolo di Sciara G 2006 The status and distribution of cetaceans in the Black Sea and Mediterranean Sea. Malaga, Spain: IUCN Centre for Mediterranean Cooperation.

[72] Rodrigues ASL, Horwitz LK, Monsarrat S, Charpentier A In press Ancient whale exploitation in the Mediterranean: species matters. Antiquity.

[73] Álvarez-Fernández E et al. 2014 Occurrence of whale barnacles in Nerja Cave (Málaga, southern Spain): Indirect evidence of whale consumption by humans in the Upper Magdalenian. Quat. Int. 337, 163–169. (10.1016/j.quaint.2013.01.014)

[74] Korsow-Richter KK, Wilson J, Jones AKG, Buckley M, van Doorn N, Collins MJ 2011 Fish ’n chips: ZooMS peptide mass fingerprinting in a 96 well plate format to identify fish bone fragments. J. Archaeol. Sci. 38, 1502–1510. (10.1016/j.jas.2011.02.014)

[75] Brown S et al. 2016 Identification of a new hominin bone from Denisova Cave, Siberia using collagen fingerprinting and mitochondrial DNA analysis. Sci. Rep. 6, 23559 (10.1038/srep23559) 27020421 PMC4810434

[76] Green RE et al. 2010 A draft sequence of the Neandertal genome. Science 328, 710–722. (10.1126/science.1188021) 20448178 PMC5100745

[77] Reich D et al. 2010 Genetic history of an archaic hominin group from Denisova Cave in Siberia. Nature 468, 1053–1060. (10.1038/nature09710) 21179161 PMC4306417

[78] Blow MJ, Zhang T, Woyke T, Speller CF, Krivoshapkin A, Yang DY, Derevianko A, Rubin EM 2008 Identification of ancient remains through genomic sequencing. Genome Res. 18, 1347–1353. (10.1101/gr.076091.108) 18426903 PMC2493433

[79] Jones MR, Good JM 2016 Targeted capture in evolutionary and ecological genomics. Mol. Ecol. 25, 185–202. (10.1111/mec.13304) 26137993 PMC4823023

[80] Briggs AW et al. 2009 Targeted retrieval and analysis of five Neandertal mtDNA genomes. Science 325, 318–321. (10.1126/science.1174462) 19608918

[81] Thalmann O et al. 2013 Complete mitochondrial genomes of ancient canids suggest a European origin of domestic dogs. Science 342, 871–874. (10.1126/science.1243650) 24233726

[82] Avila-Arcos MC et al. 2011 Application and comparison of large-scale solution-based DNA capture-enrichment methods on ancient DNA. Sci. Rep. 1, 74 (10.1038/srep00074) 22355593 PMC3216561

[83] Dabney J et al. 2013 Complete mitochondrial genome sequence of a Middle Pleistocene cave bear reconstructed from ultrashort DNA fragments. Proc. Natl Acad. Sci. USA 110, 15 758–15 763. (10.1073/pnas.1314445110) PMC3785785 24019490

[84] Meyer M et al. 2014 A mitochondrial genome sequence of a hominin from Sima de los Huesos. Nature 505, 403–406. (10.1038/nature12788) 24305051

[85] Duchêne S, Archer FI, Vilstrup J, Caballero S, Morin PA 2011 Mitogenome phylogenetics: the impact of using single regions and partitioning schemes on topology, substitution rate and divergence time estimation. PLoS ONE 6, e27138 (10.1371/journal.pone.0027138) 22073275 PMC3206919

[86] Mueller RL 2006 Evolutionary rates, divergence dates, and the performance of mitochondrial genes in Bayesian phylogenetic analysis. Syst. Biol. 55, 289–300. (10.1080/10635150500541672) 16611600

[87] Godden GT et al. 2012 Making next-generation sequencing work for you: approaches and practical considerations for marker development and phylogenetics. Plant Ecol. Divers. 5, 427–450. (10.1080/17550874.2012.745909)

[88] Slon V, Glocke I, Barkai R, Gopher A, Hershkovitz I, Meyer M 2016 Mammalian mitochondrial capture, a tool for rapid screening of DNA preservation in faunal and undiagnostic remains, and its application to Middle Pleistocene specimens from Qesem Cave (Israel). Quat. Int. 398, 210–218. (10.1016/j.quaint.2015.03.039)

[89] Hancock-Hanser BL, Frey A, Leslie MS, Dutton PH, Archer FI, Morin PA 2013 Targeted multiplex next-generation sequencing: advances in techniques of mitochondrial and nuclear DNA sequencing for population genomics. Mol. Ecol. Resour. 13, 254–268. (10.1111/1755-0998.12059) 23351075

[90] Mayer C et al. 2016 BaitFisher: a software package for multi-species target DNA enrichment probe design. Mol. Biol. Evol. (10.1093/molbev/msw056) 27009209

[91] Morin PA et al. 2010 Complete mitochondrial genome phylogeographic analysis of killer whales (*Orcinus orca*) indicates multiple species. Genome Res. 20, 908–916. (10.1101/gr.102954.109) 20413674 PMC2892092

[92] Vilstrup JT et al. 2011 Mitogenomic phylogenetic analyses of the Delphinidae with an emphasis on the Globicephalinae. BMC Evol. Biol. 11, 65 (10.1186/1471-2148-11-65) 21392378 PMC3065423

[93] Arnason U, Gullberg A, Janke A 2004 Mitogenomic analyses provide new insights into cetacean origin and evolution. Gene 333, 27–34. (10.1016/j.gene.2004.02.010) 15177677

[94] Xiong Y, Brandley MC, Xu S, Zhou K, Yang G 2009 Seven new dolphin mitochondrial genomes and a time-calibrated phylogeny of whales. BMC Evol. Biol. 9, 20 (10.1186/1471-2148-9-20) 19166626 PMC2656474

[95] Alexander A, Steel D, Slikas B, Hoekzema K, Carraher C, Parks M, Cronn R, Baker CS 2013 Low diversity in the mitogenome of sperm whales revealed by next-generation sequencing. Genome Biol. Evol. 5, 113–129. (10.1093/gbe/evs126) 23254394 PMC3595033

[96] Morin PA et al. 2015 Geographic and temporal dynamics of a global radiation and diversification in the killer whale. Mol. Ecol. 24, 3964–3979. (10.1111/mec.13284) 26087773

[97] Fernández R et al. 2016 A genomewide catalogue of single nucleotide polymorphisms in white-beaked and Atlantic white-sided dolphins. Mol. Ecol. Resour. 16, 266–276. (10.1111/1755-0998.12427) 25950249

[98] Zhou X et al. 2013 Baiji genomes reveal low genetic variability and new insights into secondary aquatic adaptations. Nat. Commun. 4, 2708 (10.1038/ncomms3708) 24169659 PMC3826649

[99] Springer MS et al. 2015 Interordinal gene capture, the phylogenetic position of Steller's sea cow based on molecular and morphological data, and the macroevolutionary history of Sirenia. Mol. Phylogenet. Evol. 91, 178–193. (10.1016/j.ympev.2015.05.022) 26050523

[100] Foote AD, Newton J, Ávila-Arcos MC, Kampmann M-L, Samaniego JA, Post K, Rosing-Asvid A, Sinding M-HS, Gilbert MTP 2013 Tracking niche variation over millennial timescales in sympatric killer whale lineages. Proc. R. Soc. B 280, 20131481 (10.1098/rspb.2013.1481) PMC3757978 23945688

[101] Foote AD, Hofreiter M, Morin PA 2012 Ancient DNA from marine mammals: studying long-lived species over ecological and evolutionary timescales. Ann. Anat. 194, 112–120. (10.1016/j.aanat.2011.04.010) 21652193

[102] Alter SE, Newsome SD, Palumbi SR 2012 Pre-whaling genetic diversity and population ecology in eastern Pacific gray whales: insights from ancient DNA and stable isotopes. PLoS ONE 7, e35039 (10.1371/journal.pone.0035039) 22590499 PMC3348926

[103] Borge T, Bachmann L, Bjørnstad G, Wiig O 2007 Genetic variation in Holocene bowhead whales from Svalbard. Mol. Ecol. 16, 2223–2235. (10.1111/j.1365-294X.2007.03287.x) 17561886

[104] Ho SYW, Saarma U, Barnett R, Haile J, Shapiro B 2008 The effect of inappropriate calibration: three case studies in molecular ecology. PLoS ONE 3, e1615 (10.1371/journal.pone.0001615) 18286172 PMC2229648

[105] Pichler FB, Baker CS 2000 Loss of genetic diversity in the endemic Hector's dolphin due to fisheries-related mortality. Proc. R. Soc. Lond. B 267, 97–102. (10.1098/rspb.2000.0972) PMC1690489 10670959

[106] Hofman CA, Rick TC, Fleischer RC, Maldonado JE 2015 Conservation archaeogenomics: ancient DNA and biodiversity in the Anthropocene. Trends Ecol. Evol. 30, 540–549. (10.1016/j.tree.2015.06.008) 26169594

