# Supplementary material for: Barcoding the largest animals on Earth: ongoing challenges and molecular solutions in the taxonomic identification of ancient cetaceans
Source: Philos Trans R Soc Lond B Biol Sci. 2016 Sep 5;371(1702):20150332. doi: 10.1098/rstb.2015.0332 (PMC4971184; doi:10.1098/rstb.2015.0332)
Supplement: Supplementary information [file rstb20150332supp1.pdf]

Supplementary material for Speller et al., 2016, Barcoding the largest animals on the earth: on-going challenges and molecular solutions in the taxonomic identification of ancient cetaceans, *Phil. Trans. R. Soc. B.* doi: 10.1098/rstb.2015-0332

Supplementary Information

1. Materials and Methods

Biomolecular analysis was applied to 17 cetacean bones recovered from seven archaeological sites, including Saint Martin, Cougourlude, and Saint Sauveur on the southern coast of France [1,2], Nuraghe Lu Brandali, Porto Torres, Villa Sant'Imbenia, in Sardinia, and San Rocchino, Tuscany, Italy [3]. Based on previous morphological analysis, four of these samples from Saint Sauveur were presumed to represent possible gray whale remains [4] while the other could not be confidently assigned to species.

1.1 DNA sample preparation, extraction and amplification

The ancient whale samples were prepared and processed for DNA extraction in the Ancient DNA laboratory at University of York, following strict protocols for contamination control and detection, including positive pressure, the use of protective clothing, UV sources for workspace decontamination, and laminar flow hoods for extraction and PCR-set-up. Fragment of bone were immersed in 6% sodium hypochlorite for 5 mins, rinsed two times in HPLC grade water, UV irradiated for 30 min on two sides, and ground into powder. DNA from 20-55 mg of bone powder was extracted using a silica spin column protocol [5] as modified in Dabney et al. [6], and DNA was eluted in 50ul. PCR amplifications targeted a 182bp fragment of cytochrome b mitochondrial gene which has been demonstrated to successfully distinguish cetacean species [7,8]. PCR reactions and cycling conditions followed those described in Speller et al. [9]; successfully amplified products were sequenced using the forward primer at Eurofins Genomics, Ebersberg, Germany.

1.2 mtDNA sequence analysis and species identifications

ChromasPro software (www.technelysium.com.au) was used to visually analyse and edit the sequences and truncate primer sequences. Sequences were compared with published references through the GenBank BLAST application (http://www.ncbi.nlm.nih.gov/BLAST/), with multiple alignments of ancient and published reference was sequences conducted using ClustalW [10], through BioEdit (http://www.mbio.ncsu.edu/BioEdit). Species identifications were assigned to a sample only if it was identical to published reference sequences from a single species in GenBank; species identities were further confirmed through 'DNA Surveillance', a web-based programme which provides robust cetacean identifications based on comparisons with a comprehensive set of validated cetacean reference sequences [11]. Twelve sequences were uploaded to the Genetic Sequence Database at the National Center for Biotechnical Information (NCBI) (GenBank ID:KT923090-KT923101).

1.3 Collagen peptide mass fingerprinting

The 17 cetacean samples were analyzed using the ZooMS protocol described in Buckley et al [12] and Evans et al. [8]. Between 10-30 mg of bone powder was fully demineralized through immersion in 0.6 M hydrochloric acid at room temperature or at 4°C. Samples WH505-507, WH511-513, and WH801-804 were centrifuged, the supernatant was discarded, and the samples rinsed three times with 200 µl AmBic solution (50 mMol ammonium bicarbonate, pH 8.0) before being gelatinised in 100 µl of AmBic solution for 1 hour at at 65˚C.

WH501-504 and WH508-510 underwent an additional ultrafiltration step. Following demineralization, these samples were were centrifuged, the supernatant was discarded, and the collagen gelatinised through incubation in 250 µl of 0.6M HCl for three hours at 65˚C. The collagen was ultrafiltered using Amicon Ultra-4 centrifugal filter units (30,000NMWL, EMD Millipore) until the supernatant was concentrated to approximately 100 µl. The retentate was washed three times with 200 µl AmBic solution, and concentrated to a final volume of 50 µl.

For all samples, the resulting collagen was incubated with 0.4µg of trypsin overnight at 37˚C, acidified to 0.1% trifluoroacetic acid (TFA). The collagen was purified using a 100 µl C18 resin ZipTip® pipette

tip (EMD Millipore) with conditioning and eluting solutions composed of 50% acetonitrile and 0.1% TFA, while 0.1% TFA was used for the lower hydrophobicity buffer. The resulting collagen was eluted in 50 µl.

1.4 Mass spectrometry and taxonomic identifications

One microlitre of the collagen extract was mixed with 1 µl of α-cyano-hydroxycinnamic acid matrix solution (1% in conditioning solution) and spotted onto a 384 spot MALDI target plate, with calibration standards. Sample were spotted in triplicate, and run on a Bruker ultraflex III MALDI TOF/TOF mass spectrometer with a Nd:YAG smart beam laser. A SNAP averaging algorithm was used to obtain moniosotopic masses (C 4.9384, N 1.3577, O 1.4773, S 0.0417, H 7.7583), resulting in a total of 51 individual spectra.

mMass software [13] was used to visually inspect the spectra; spectra from replicates of the same sample were averaged, and compared to the list of *m/z* markers for marine mammals presented in Buckley et al. [14] and Kirby et al. [15]. Taxonomic identifications were assigned at the most conservative level of identification (genus, or family level) based on the presence of unambiguous *m/z* markers.

2. Results

2.1 Taxonomic identifications

Following analysis of the mtDNA sequences and PMF spectra, taxonomic identifications could be assigned to 15 of the 17 samples. Taxonomic identifications were assigned to 12 archaeological samples using ancient mtDNA sequences and 14 samples using PMF spectra (Table S2; Table S3). The combined results produced 11 fin whale (*Balaenoptera physalus*), one sperm whale (*Physeter catodon*), one right whale (*Eubalaena glacialis*), one Cuvier's beaked whale (*Ziphius cavirostris*) and one family level identification (*Mysticeti*). ZooMS and mtDNA identifications were consistent for the 11 samples which produced results using both methods. The three samples that failed to amplify using the whale-specific cytb primers (WH502, 504, 509), also failed to produce unambiguous ZooMS identifications, suggesting poor overall biomolecular preservation in these samples.

© The Authors under the terms of the Creative Commons Attribution License http://creativecommons.org/licenses/by/3.0/, which permits unrestricted use, provided the original author and source are credited.

Supplementary Figure 1: Averaged MALDI-ToF mass spectra from sample WH509, which was identified only to the level of Mysticeti based on a lack of high-weight molecular markers necessary to differentiate fin whale, gray whale and humpback whale (Buckley et al. 2014)

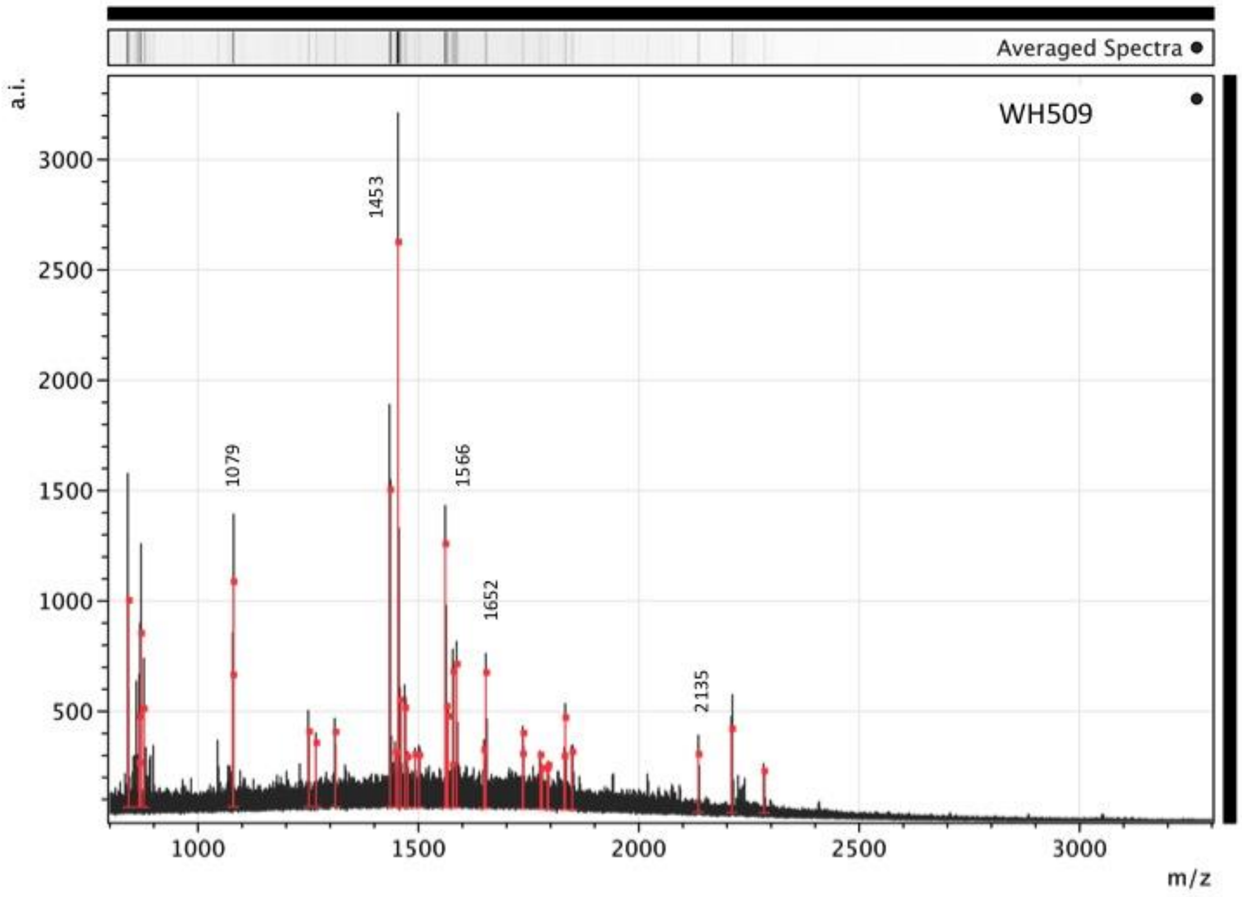

© The Authors under the terms of the Creative Commons Attribution License http://creativecommons.org/licenses/by/3.0/, which permits unrestricted use, provided the original author and source are credited.

Supplementary Table 1: Species Identification summary of archaeological cetaceans remains from the North Sea Case study

Species	Skull Fragment	Maxilla	Mandible	Cervical Vertebra	Thoracic Vertebra	Lumbar Vertebra	Caudal Vertebra	Vertebra	Vertebral Epiphysis	Rib	Scapula	Humerus	Radius	Ulna	Phalanx	Partial Skeleton	Unidentified	Element not specified	TOTAL
Unknown Cetacean	1		3	1				14		1	1						201		**221**
**Common Bottlenose Dolphin**	2		1		12	8	9	2			2	1						136	**173**
**Harbour Porpoise**	12		1		3	7	13	16		1	2							32	**87**
Common Bottlenose Dolphin Cf.																		50	**50**
Large Cetacean								9		1									**10**
**White Beaked Dolphin**							1											9	**10**
**Sperm Whale**							1	3		2								3	**9**
**Killer Whale**					1	1	2		1			1	1	1				1	**9**
Unknown Baleen Whale	1		2							1							4		**8**
**Northern Atlantic Right Whale**	1						1	2								2			**6**
Unknown Dolphin	1							3									1		**5**
Unknown Odontoceti				3							1								**4**
**Northern Bottlenose Whale**							2	1											**3**
**Common Minke Whale**																		3	**3**
Common Minke Whale Cf.																		3	**3**
Unknown Beaked Whale								2											**2**
**Long Finned Pilot Whale**										1		1							**2**
**Fin Whale**										1								1	**2**
Blue/Fin Whale												2							**2**
Large Odontoceti																	1		**1**
**Short Beaked Common Dolphin**																		1	**1**
Common Bottlenose Dolphin/White Beaked Dolphin		1																	**1**
Long Finned Pilot Whale Cf.								1											**1**
**Humpback Whale**															1				**1**
Unknown Rorqual																	1		**1**
Sperm/Right/Humpback Whale						1													**1**
**TOTAL**	**18**	**1**	**7**	**4**	**16**	**17**	**29**	**53**	**1**	**8**	**6**	**5**	**1**	**1**	**1**	**2**	**208**	**239**	**616**

© The Authors under the terms of the Creative Commons Attribution License http://creativecommons.org/licenses/by/3.0/, which permits unrestricted use, provided the original author and source are credited.

Supplementary Table 2: Sample information, mtDNA and collagen PMF (ZooMS) results for the archaeological whale bones

Lab Code	Archaeological site	Date	Evidence of processing	Morphological Identification	Bone	Fragment	Reference	DNA species ID	Collagen PMF (ZooMS) ID
WH501	Saint Martin, France	Late antiquity	carcass processing activity	Balaenidae	lumbar vertebra	processus lateral		E. glacialis	Right whale
WH502	Cougourlude, France	Roman	manufactured object		long bone	diaphysis		no amplification	Poor Spectra
WH503	Cougourlude, France	Roman	carcass processing activity	Balaenoptera	thoracic vertebra	processus dorsal		B. physalus	Poor Spectra
WH504	Cougourlude, France	Roman	carcass processing activity		lumbar vertebra	processus lateral		no amplification	Poor Spectra
WH505	Saint Sauveur, France	Roman	manufactured object	Eschrichtius or Balaenoptera	thoracic vertebra	body, C2 or C6	Macé 2003, fig 2	B. physalus	Fin Whale
WH506	Saint Sauveur, France	Iron Age II	no evidence			undetermined		B. physalus	Fin whale
WH507	Saint Sauveur, France	Iron Age II	carcass processing activity	Eschrichtius or Balaenoptera	vertebra	body	Macé 2003, fig. 3	B. physalus	Fin whale
WH508	Saint Sauveur, France	Iron Age II	carcass processing activity	Balaenidae or Eschrichtidae	rib	body	Macé 2003, fig. 5	P. catodon	Sperm Whale
WH509	Saint Sauveur, France	Iron Age II	no evidence	Eschrichtius or Megaptera	maxillar (upper)	blowhole fragment	Macé 2003, fig 4	no amplification	Mysticeti
WH510	Saint Sauveur, France	Late Antiquity/Early Middle Age	manufactured object	Eschrichtius	cervical vertebra	body	Macé 2003, fig 1	B. physalus	Fin whale
WH511	Saint Sauveur, France	Iron Age II	no evidence		vertebra	fragment		B. physalus	Fin whale
WH512	Saint Sauveur, France	Iron Age II	no evidence		unknown	undetermined		B. physalus	Fin whale
WH513	Saint Sauveur, France	Iron Age II	no evidence		unknown	undetermined		B. physalus	Fin whale
WH801	Nuraghe Lu Brandali, Sardinia	Bronze Age	Butchery marks		Vertebra	body		Z. cavirostris	Beaked Whale
WH802	Porto Torres, Sardinia	Roman	Butchery marks		unknown			no amplification	Fin whale
WH803	Villa Sant'Imbenia, Sardinia	Middle Ages (C7-8 AD)	Butchery marks		Scapula	?		B. physalus	Fin whale
WH804	San Rocchino, Italy	Iron Age	Butchery marks		Vetebra	body		no amplification	Fin whale

Supplementary Table 3: Designated collagen peptide markers used for taxonomic identification of the archaeological cetacean samples

Sample No.	(P1)	α2(I) 988–1000 (A)	α2(I) 494–508 (B)	α2(I) 512–529 (C)	(P2)	α2(I) 803–826 (D)	α1(I) 602–634 (F)	α2(I) 767–799 (G)	Collagen PMF ID
**WH501**	1079	1205?	1453	1566	1682	2135	2883	3023	Right whale
**WH502**	-	-	-	-	-	-	-	-	No identification
**WH503**	-	-	-	-	-	-	-	-	No identification
**WH504**	-	-	1453	-	1652	-	-	-	No identification
**WH505**	1079	1205?	1453	1566?	1652	2135	2883	3023	Fin Whale
**WH506**	1079	-	1453	-	1652	2135	2883	3023	Fin whale
**WH507**	1079	-	1453	1566?	1652	2135	2883	3023	Fin whale
**WH508**	1079	1205	1453	1550	1652	2133	2883	-	Sperm Whale
**WH509**	1079	-	1453	1566	1652	2135	2883?	-	Mysticeti
**WH510**	1,079	1,205	1,453	1,566	1,652	2,135	2,883	-	Fin whale
**WH511**	1079	1205?	1453	1566?	1652	2135	2883	3023	Fin whale
**WH512**	1079	1205	1453	1566?	1652	2135	2883	3023	Fin whale
**WH513**	1079	1205	1453	1566?	1652	2135	2883	3023	Fin whale
**WH801**	1063	-	1441	1550	-	2091?	-	-	Beaked whale
**WH802**	1079	1205	1453	1566?	1652	2135	2883	3023	Fin whale
**WH803**	1079	1205	1453	1566?	1652	2135	2883	3023	Fin whale
**WH804**	1079	1205	1453	1566?	1652	2135	2883	3023	Fin whale

? Indicates peak is present but at low intensity, or below signal to noise threshold

© The Authors under the terms of the Creative Commons Attribution License http://creativecommons.org/licenses/by/3.0/, which permits unrestricted use, provided the original author and source are credited.

References:

1. Py, M. 2009 *Lattara, Lattes, Hérault. Comptoir gaulois méditerranéen entre Étrusques, Grecs, et Romains*. Éditions Errance.

2. Duperron, G. 2012 L'établissement antique de Saint-Martin-Le-Bas à Gruissan (Aude). In *Les ports antiques de Narbonne, vol. 2* (ed C. Sanchez), Programme Collectif de Recherche, Ministère de la Culture, UMR 5140.

3. Wilkens, B. 2003 La Fauna sarda durante l'Olocene: le conoscenze attuali. *Sardinia, Corsica et Baleares Antiquae* **1**, 181–197.

4. Macé, M. 2003 Did the Gray whale, Eschrichtius robustus, calve in the Mediterranean? *Lattara* **16**, 153–164.

5. Yang, D. Y., Eng, B., Waye, J. S., Dudar, J. C. & Saunders, S. R. 1998 Technical note: improved DNA extraction from ancient bones using silica-based spin columns. *Am. J. Phys. Anthropol.* **105**, 539–543.

6. Dabney, J. et al. 2013 Complete mitochondrial genome sequence of a Middle Pleistocene cave bear reconstructed from ultrashort DNA fragments. *Proc. Natl. Acad. Sci. U. S. A.* **110**, 15758–15763. (doi:10.1073/pnas.1314445110)

7. Yang, D. Y. & Speller, C. F. 2006 Co-amplification of cytochrome b and D-loop mtDNA fragments for the identification of degraded DNA samples. *Mol. Ecol. Notes* **6**, 605–608.

8. Evans, S. et al. 2016 Using combined biomolecular methods to explore whale exploitation and social aggregation in hunter–gatherer–fisher society in Tierra del Fuego. *J. Archaeol. Sci. Rep.* 6, 757–767.

9. Speller, C. F., Hauser, L., Lepofsky, D., Moore, J., Rodrigues, A. T., Moss, M. L., McKechnie, I. & Yang, D. Y. 2012 High potential for using DNA from ancient herring bones to inform modern fisheries management and conservation. *PLoS One* **7**, e51122. (doi:10.1371/journal.pone.0051122)

10. Thompson, J. D., Higgins, D. G. & Gibson, T. J. 1994 CLUSTAL W: improving the sensitivity of progressive multiple sequence alignment through sequence weighting, position-specific gap penalties and weight matrix choice. *Nucleic Acids Res.* **22**, 4673–4680. (doi:10.1093/nar/22.22.4673)

11. Baker, C. S., Dalebout, M. L., Lavery, S. & Ross, H. A. 2003 www.DNA-surveillance: applied molecular taxonomy for species conservation and discovery. *Trends Ecol. Evol.* **18**, 271–272. (doi:10.1016/S0169-5347(03)00101-0)

12. Buckley, M., Collins, M., Thomas-Oates, J. & Wilson, J. C. 2009 Species identification by analysis of bone collagen using matrix-assisted laser desorption/ionisation time-of-flight mass spectrometry. *Rapid Commun. Mass Spectrom.* **23**, 3843–3854. (doi:10.1002/rcm.4316)

13. Strohalm, M., Hassman, M., Kosata, B. & Kodícek, M. 2008 mMass data miner: an open source alternative for mass spectrometric data analysis. *Rapid Commun. Mass Spectrom.* **22**, 905–908. (doi:10.1002/rcm.3444)

14. Buckley, M., Fraser, S., Herman, J., Melton, N., Mulville, J. & Pálsdóttir, A. 2014 Species identification of archaeological marine mammals using collagen fingerprinting. *J. Archaeol. Sci.* **41**, 631–641. (doi:10.1016/j.jas.2013.08.021)

15. Kirby, D. P., Buckley, M., Promise, E., Trauger, S. A. & Holdcraft, T. R. 2013 Identification of collagen-based materials in cultural heritage. *Analyst* **138**, 4849–4858. (doi:10.1039/c3an00925d)
